# Identification of a Novel *MLPH* Missense Mutation in a Chinese Griscelli Syndrome 3 Patient

**DOI:** 10.3389/fmed.2022.896943

**Published:** 2022-05-06

**Authors:** Qiaorong Huang, Yefeng Yuan, Juanjuan Gong, Tianjiao Zhang, Zhan Qi, Xiumin Yang, Wei Li, Aihua Wei

**Affiliations:** ^1^Department of Dermatology, Beijing Tongren Hospital, Capital Medical University, Beijing, China; ^2^Beijing Key Laboratory for Genetics of Birth Defects, Beijing Pediatric Research Institute, Beijing, China; ^3^Rare Disease Center, National Center for Children's Health, Beijing, China; ^4^MOE Key Laboratory of Major Diseases in Children, Beijing, China; ^5^Beijing Children's Hospital, Capital Medical University, Beijing, China

**Keywords:** Griscelli syndrome, MLPH, melanosome, pathogenic variant, hypopigmentation

## Abstract

Melanophilin (MLPH) functions as a linker between RAB27A and myosin Va (MYO5A) in regulating skin pigmentation during the melanosome transport process. The MYO5A-MLPH-RAB27A ternary protein complex is required for anchoring mature melanosomes in the peripheral actin filaments of melanocytes for subsequent transfer to adjacent keratinocytes. Griscelli syndrome type 3 (GS3) is caused by mutations in the *MLPH* gene. So far, only five variants of *MLPH* associated with GS3 have been reported. Here, we reported the first patient with GS3 in a Chinese population. The proband carried a novel homozygous missense mutation (c.73G>C; p.D25H), residing in the conserved Slp homology domain of MLPH, and presented with hypopigmentation of the hair, eyebrows, and eyelashes. Light microscopy revealed the presence of abnormal pigment clumping in his hair shaft. *In silico* tools predicted this *MLPH* variant to be likely pathogenic. Using immunoblotting and immunofluorescence analysis, we demonstrated that the MLPH (D25H) variant had an inhibitory effect on melanosome transport by exhibiting perinuclear melanosome aggregation in melanocytes, and greatly reduced its binding to RAB27A, although the protein level of MLPH in the patient was not changed. Our findings suggest that MLPH (D25H) is a pathogenic variant that expands the genetic spectrum of the *MLPH* gene.

## Introduction

Melanosomes are intracellular lysosome-related organelles (LRO) in which melanin is synthesized, stored, and transported (1, 2). After maturation in the perinuclear region of melanocytes, melanosomes are transported to the cell periphery and dendritic tips by coordinating bi-directional transport on microtubules and anterograde transport on actin filaments (3, 4). Melanophilin (MLPH), myosin Va (MYO5A), and RAB27A form a tripartite complex involved in melanosome transport along with the microtubules and actin-network (5, 6). Of these, MLPH has a critical role in bridging RAB27A on the melanosomes and MYO5A on the actin filaments during melanosome transport (7). Mutations of any subunit of the complex, MYO5A, RAB27A, and MLPH cause the rare autosomal recessive inherited disease, Griscelli syndrome (GS) types 1~3 (8–10).

All patients with GS1~3 present relatively mild hypopigmentation in their hair and skin. GS3 (*MLPH* mutations) is restricted to a hypopigmentation disorder, while GS1 (*MYO5A* mutations) and GS2 (*RAB27A* mutations) additionally exhibit neurological dysfunctions or immunological defects. Patients with GS3 are very rare compared with GS1 and GS2 (11). To date, there are only five variants of *MLPH* associated with GS3 recorded in the Human Gene Mutation Database (8, 11–13) (HGMD, version 2021.10) or reported in the literature (14).

In this study, we identified a novel *MLPH* p.D25H mutation in a Chinese GS3 non-consanguineous family, and we provide evidence that this *MLPH* missense mutation leads to aberrant melanosome transport in melanocytes.

## Materials and Methods

### Patient Information

A 32-year-old male patient from the Chinese Han population had unexplained pigmentary dilution of the hair, eyebrows, and eyelashes. He visited Beijing Tongren Hospital, Capital Medical University in June 2021 and was recruited for this study. Blood samples were obtained from the patient and his parents. This study was approved by the ethics committees of Beijing Tongren Hospital and Beijing Children's Hospital, Capital Medical University. Written informed consent was obtained in accordance with the declaration of Helsinki.

### Genetic Analysis

Whole exome sequencing was performed on genomic DNA from the proband. The Agilent SureSelect Human All ExonV6 Kit (Agilent Technologies, Santa Clara, CA, USA) was used to target the exonic regions of the genome. The Illumina NovaSeq 6000 platform (Illumina Inc., San Diego, CA, USA) was used for genomic DNA sequencing by Novogene Bioinformatics Technology Co., Ltd (Beijing, China) to generate 150 bp paired-end reads with a minimum coverage of 20× for ~95% of the genome (mean coverage of ~100×). The DNA sequences were analyzed by in-house quality control software to remove low-quality reads and were then aligned to the reference human genome (hs37d5) using the Burrows-Wheeler Aligner (BWA) (15), and duplicate reads were marked using Sambamba tools (16). Single nucleotide variants (SNVs) and indels were called by GATK to generate a gVCF file. The sequence variants in the proband and his parental samples were confirmed by Sanger sequencing analysis.

### Homology Analysis and Structural Modeling of MLPH

The human MLPH protein (NP_077006.1) sequence was aligned for analysis of the conservation of the mutated residue (p.D25H) with the sequences of the following homologous proteins: *Mus musculus* (NP_443748.2), *Felis catus* (NP_001073123.1), *Ovis aries* (NP_001139743.1), *Oryctolagus cuniculus* (NP_001284414.1), *Canis lupus familiaris* (NP_001096689.2), *Rattus norvegicus* (NP_001012135.1), and *Gallus* (NP_001108552.1). Conservation analysis and alignment visualization were performed by Clustal Omega (http://www.clustal.org/omega/) and Jalview software (17). The protein structure was drawn using the online tool Illustrator for Biological Sequences (IBS) (http://ibs.biocuckoo.org/). The domains of human MLPH protein referred to the structure of mouse Slac2-a/melanophilin protein (18).

### Plasmid Construction

The full-length cDNA of human *MLPH* with C terminal GFPSpark tag was synthesized by Sino Biological Inc. The human entire coding region of *MLPH* was subcloned into the pEGFP-C2 vector with an EGFP-tag and the pCMV-tag2B vector with a Flag-tag. The human *MLPH* sequence encoding the first 146 amino acids, termed Slp homology domain (SHD) (19), was subcloned into the pCMV-tag3B vector with a Myc-tag. The full-length cDNA of human RAB27A was amplified from total RNA of the human melanoma cell line MNT-1 cells (ATCC, USA) by one-step RT-PCR and the digested PCR product was cloned into the pCMV-tag2B vector with a Flag-tag. To introduce the point mutations into the *MLPH* or *SHD*, we used site-directed mutagenesis primers and high-fidelity polymerase to amplify the entire plasmid by PCR. The primers used were as follows: 5′-GTCTTGGAAGTTGTTCAACGACATTTTGACCTCCGAAGGAAAG-3′ (D25H primer; sense); 5′-CTTTCCTTCGGAGGTCAAAATGTCGTTGAACAACTTCCAAGAC-3′ (D25H primer; antisense); 5′-CGAAGGAAAGAAGAGGAATGGCTAGAGGCGTTGAAG-3′ (R35W primer; sense); and 5′-CTTCAACGCCTCTAGCCATTCCTCTTCTTTCCTTCG-3′ (R35W primer; antisense).

### Cell Culture and Transfection

Briefly, MNT-1 cells (ATCC, USA) were cultured in minimal essential medium (MEM) supplemented with 20% fetal bovine serum (FBS, Invitrogen), 10% AIM-V medium (Gibco), sodium pyruvate, and non-essential amino acids at 37°C with 5% CO_2_ (20). Human embryonic kidney 293 T (HEK293T) cells were cultured in DMEM (Gibco) with 10% FBS at 37°C with 5% CO_2_. Subsequently, FLAG-MLPH or GFP-MLPH wild-type (WT) and MLPH mutant (p.D25H) expression plasmids were transfected into the cells using Lipofectamine 3000 (Invitrogen) in Opti-MEM (Gibco). After 6 h, the medium was changed to a fresh medium for further experiments.

### Western Blotting

A total of 2 ml of blood was collected from each individual in sodium citrate blood collection tubes. Platelet-rich plasma was obtained by centrifugation at 150 g for 10 min at room temperature. Washed platelets were lysed in a lysis buffer (50 mM Tris pH 7.4, 150 mM NaCl, 0.1% sodium dodecyl-sulfate (SDS), 1% Triton X-100, and 1% sodium deoxycholate) with a protease inhibitor cocktail (Sigma-Aldrich, P8340) mixture, boiled with 5× loading buffer and then separated by 10% SDS polyacrylamide gel electrophoresis (SDS-PAGE) (21). Samples were then transferred to a polyvinylidene difluoride (PVDF) membrane (Millipore). The membranes were blocked with 5% milk in PBS (0.1% Tween) for 1 h. Primary antibodies, anti-MLPH (Proteintech, 10338-1-AP, 1:5,000), anti-MYO5A (Cell Signaling Technology, 34025, 1:1,000), anti-RAB27A (Santa Cruz Biotechnology, sc-81914, 1:2,000), and anti-glyceraldehyde-3-phosphate dehydrogenase (anti-GAPDH) (Cell Signaling Technology, 5174T, 1:5,000), were incubated overnight at 4°C. After washing with PBS-T, membranes were incubated with a secondary antibody (goat anti-mouse IgG or goat anti-rabbit IgG, 1:5,000; Invitrogen) for 2 h at room temperature, then developed with the ECL substrate (Thermo Scientific).

### Immunofluorescence Staining and Confocal Imaging

In brief, MNT-1 cells transfected with GFP-MLPH (WT) or GFP-MLPH (D25H) were grown for 24 h on coverslips. Cells were fixed with 4% paraformaldehyde for 10 min, washed with PBS, permeabilized in 0.1% Triton X-100/PBS for 10 min, and blocked in 1% bovine serum albumin (BSA)/PBS for 1 h. Coverslips were then incubated with mouse anti-TYRP1 (Covance, SIG-38150) diluted 1:500 in 1% BSA/PBS at 4°C overnight. Then, cells were washed and incubated for 2 h with a 1:500 dilution with donkey anti-mouse secondary antibody conjugated to ALEXA-594 (Invitrogen) at room temperature. After washes, cells were mounted in 4',6-diamidino-2-phenylindole (DAPI, ZSGB-BIO). Finally, confocal images were acquired using the Zeiss LSM 880 Confocal Microscope (German).

### Co-immunoprecipitation (Co-iP) Assays

Transfected HEK293T cells with Flag-tagged pCMV-tag2B-RAB27A and Myc-tagged pCMV-tag3B-SHD (WT, D25H, or R35W) were harvested after 48 h, then were incubated with a lysis buffer (150 mM NaCl, 50 mM Tris pH 7.4, 1 mM EDTA, and 1% Triton-100) with a protease inhibitor cocktail for 30 min at 4°C. Lysates were centrifuged at 12,000× g for 10 min at 4°C and 10% of the whole-cell lysates were taken as inputs. The remaining lysates were incubated with pre-washed anti-FLAG M2-Agarose affinity gel (Sigma-Aldrich, FLAGIPT-1) at 4°C, overnight. The bead complexes were washed 5 times with a washing buffer (150 mM NaCl and 50 mM Tris pH 7.4), and the proteins were eluted in a 2× loading sample buffer and then subjected to 10% SDS-PAGE for western blotting according to the procedures in our previous study (21).

## Results

### Clinical Findings

**Figure 1:**
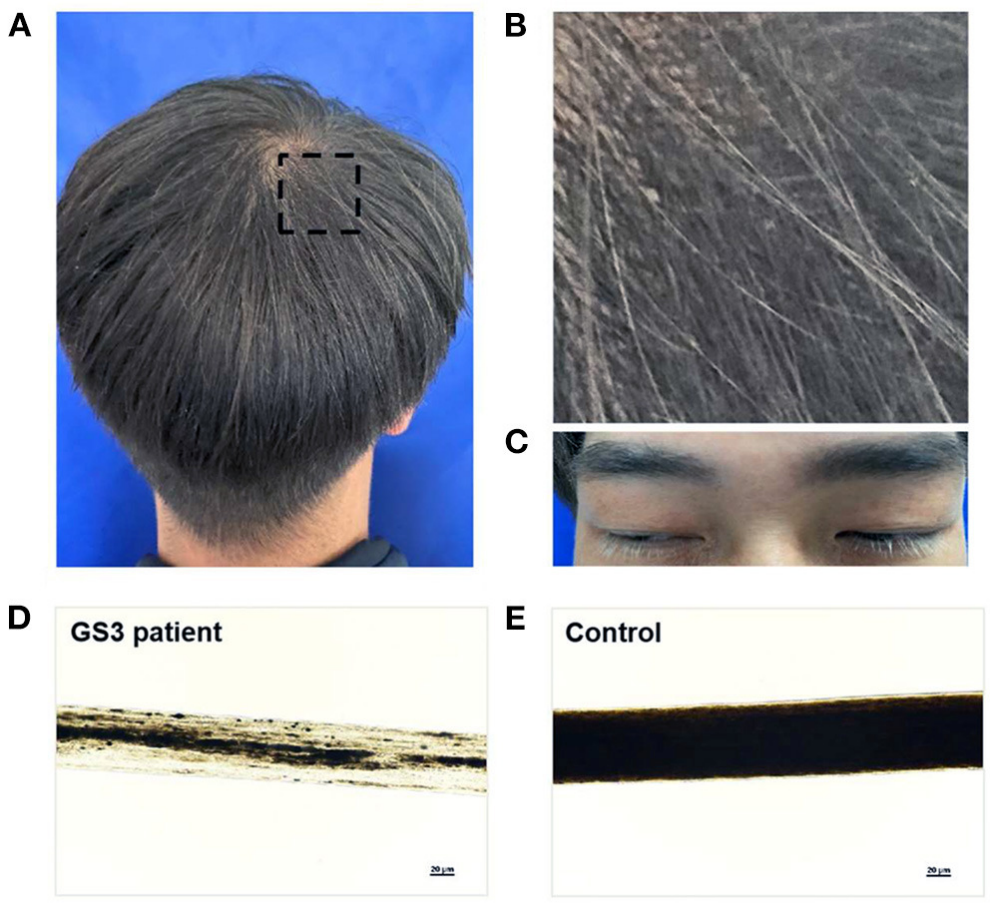
Hair phenotypes of a patient with Griscelli syndrome type 3 (GS3). **(A,B)** A mixture of dark brown and silvery-gray hair in a 32-year-old man with GS3. The black rectangular frame is enlarged in **(B)**. **(C)** Dark gray eyebrows and white eyelashes of the patient. **(D,E)** Light microscopy images of the hair shafts, showing the irregular clumping of melanin in the patient's hair shaft **(D)**, and the even distribution of melanin in the hair shaft from a normal black hair individual **(E)**. The magnification of the objective lenses was×20. Written informed consent was obtained from the patient for publication of the pictures **(A–C)**.

The proband was a male patient aged 32 years at the time of the examination. He had brownish and silvery-gray hair, dark gray eyebrows, and white eyelashes (Figures 1A–C). His hair shaft showed uneven melanin granules under light microscopy (Figure 1D), a typical feature observed in GS3 (22). Blood parameters, such as hemoglobin, white blood cell (WBC), and platelets were within normal ranges. The value of erythrocyte sedimentation rate (ESR) was 3 mm/h (normal range, 0~15 mm/h). Specific immunoglobulin levels (IgG, IgM, and IgA) were normal. Complement 3 (C3) was 89.3 mg/dl (normal range, 90~180 mg/dl) and C4 was normal. The ADP-induced platelet aggregation of 43.6% (normal range, 59.1~98.3%) was lower than normal. Several coagulation tests, such as prothrombin time (PT) of 11.8 s (normal range, 10.5~15 s), activated partial thromboplastin time (APTT) of 27.2 s (normal range, 22.7~31.8 s), and thrombin time (TT) of 17.5 s (normal range, 14~21 s), were within normal limits. Fibrinogen (FIB) of 1.89 g/L (normal range, 2~4 g/L) was slightly lower than normal. Brain MRI was normal. Visual acuity was 20/20 in both eyes. The fundus photograph (Supplementary Figure S1A), the anterior segment picture (Supplementary Figure S1B), and the optical coherence tomography (OCT) (Supplementary Figure S1C) were normal.

### Identification of a Novel *MLPH* Mutation

**Figure 2:**
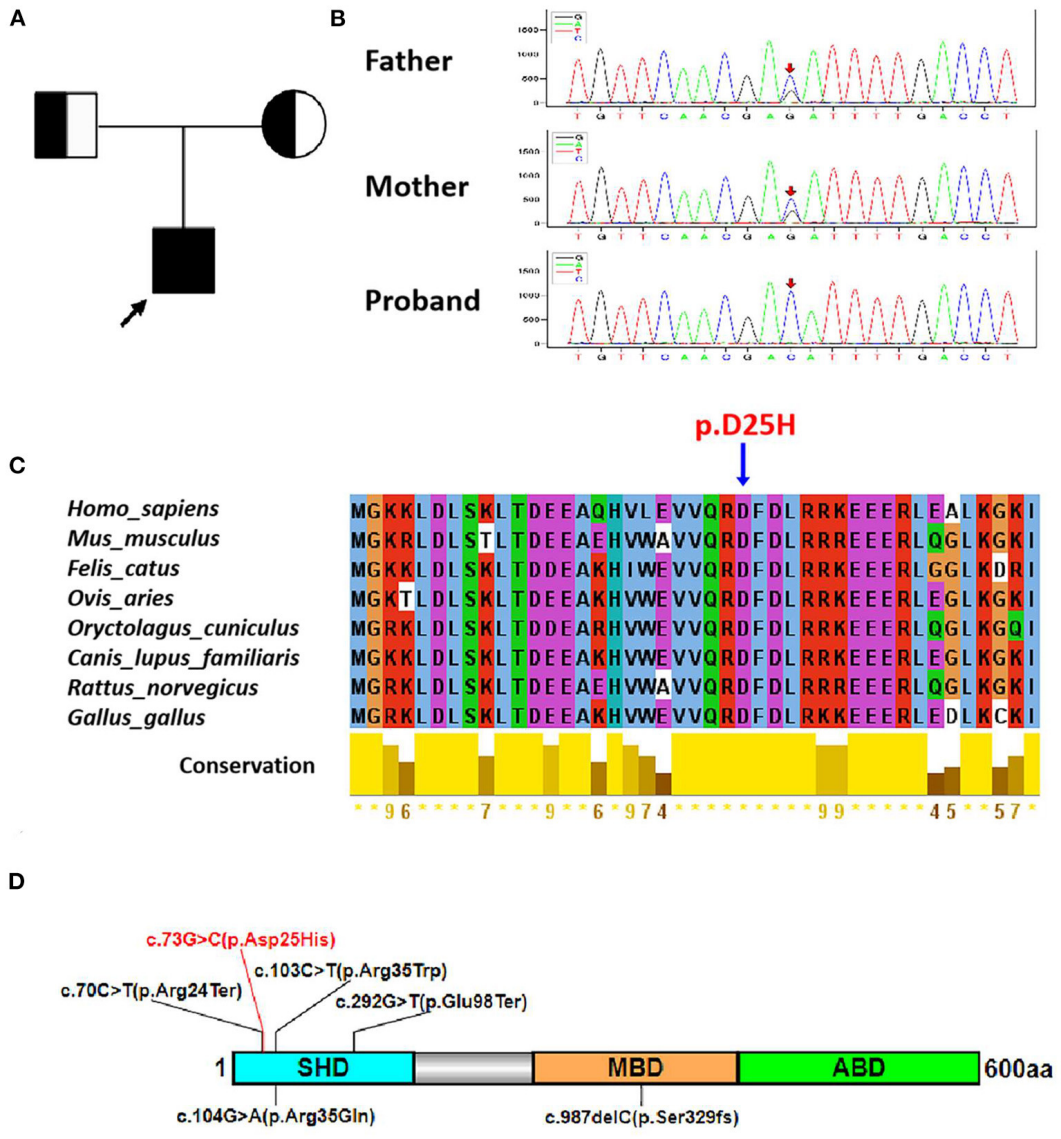
The p.D25H variant in the patient with GS3. **(A)** A pedigree of the GS3 family. The black arrow indicates the proband. **(B)** Sanger sequencing analysis shows the homozygous c.73G>C mutation in proband, the heterozygous c.73G>C mutation in the parents of the patient. Red arrows show the mutational site. **(C)** Sequence alignment of MLPH protein in different species. The blue arrow indicates the mutational site. **(D)** Schematic representation of human MLPH protein structure. The novel missense mutation in this study and other reported mutations in MLPH are represented by red and black lines, respectively. SHD, Slp homology domain; MBD, MYO5A-binding domain; ABD, actin-binding domain.

Mutational screening for more than 200 hypopigmentation-related genes using next-generation sequencing (NGS) technologies identified a novel homozygous missense variant c.73G>C (p. Asp25His) (RefSeq NM_024101.7) of the *MLPH* gene in the proband (Figure 2A), which was verified by Sanger sequencing (Figure 2B). Meanwhile, Sanger sequencing revealed his parents who were not consanguineous as heterozygous carriers of this mutation (Figure 2B). Using *in silico* tools, such as PROVEAN, PolyPhen-2, and Mutation Taster, we evaluated the pathological effects of the c.73G>C mutation on the function of MLPH, and the allele frequency of the c.73G>C variant was not available in three common databases (Table 1), predicting it as likely pathogenic (23). Conservation analysis of the protein sequence in different species showed that the Asp25 residue is highly conserved (Figure 2C). Six *MLPH* mutations that caused GS3, including this newly reported allele, are mainly clustered on the SHD (Figure 2D) (8, 11–14), suggesting that this region appears to be a mutational hotspot region.

**Table 1:** Effects of novel MLPH mutation predicted using *in silico* tools.

**Chromosome 2** **co-ordinates**	**cDNA** **alteration**	**Amino acid** **alteration**	**Mutation** **type**	**ExAC allele** **frequency**	**gnomAD allele** **frequency**	**1000 G** **Project**	**PROVEAN**	**PolyPhen-2**	**Mutation taster**
238402142G>C	c.73G>C	p.D25H	Missense	N/A	N/A	N/A	Deleterious	Probably damaging	Disease causing

### Expression and Localization of MLPH (D25H) Are Not Altered

**Figure 3:**
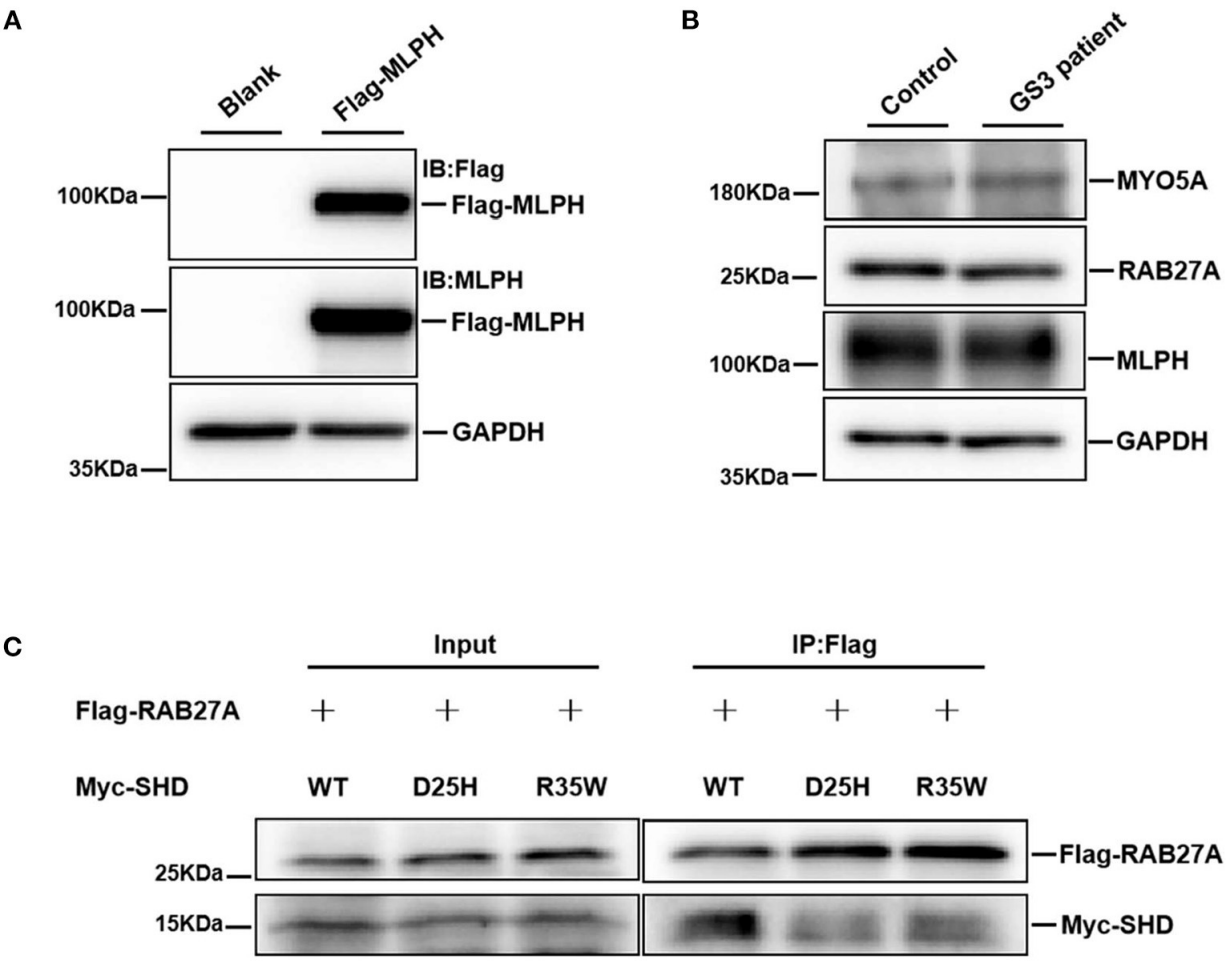
The patient with GS3 displays normal expression of endogenous MLPH, MYO5A, and RAB27A, but the mutant MLPH (D25H) decreases its interaction with RAB27A. **(A)** HEK293T cells were transfected with the Flag-MLPH construct, and immunoblotted with indicated antibodies. Glyceraldehyde-3-phosphate dehydrogenase (GAPDH) serves as a loading control. The immunoblotting data show both Flag and MLPH antibody recognized the same specific band. **(B)** Western blotting analysis of protein extracts from peripheral platelets of a healthy control and the GS3 patient with indicated antibodies. There were no apparent differences in protein levels of MLPH, MYO5A, and RAB27A between control and patient. **(C)** Co-immunoprecipitation of Flag-RAB27A with Myc-SHD. SHD (R35W)-MLPH served as a positive control. Both SHD (D25H)-MLPH and SHD (R35W)-MLPH decreased the interaction with RAB27A. These experiments were repeated three independent times.

**Figure 4:**
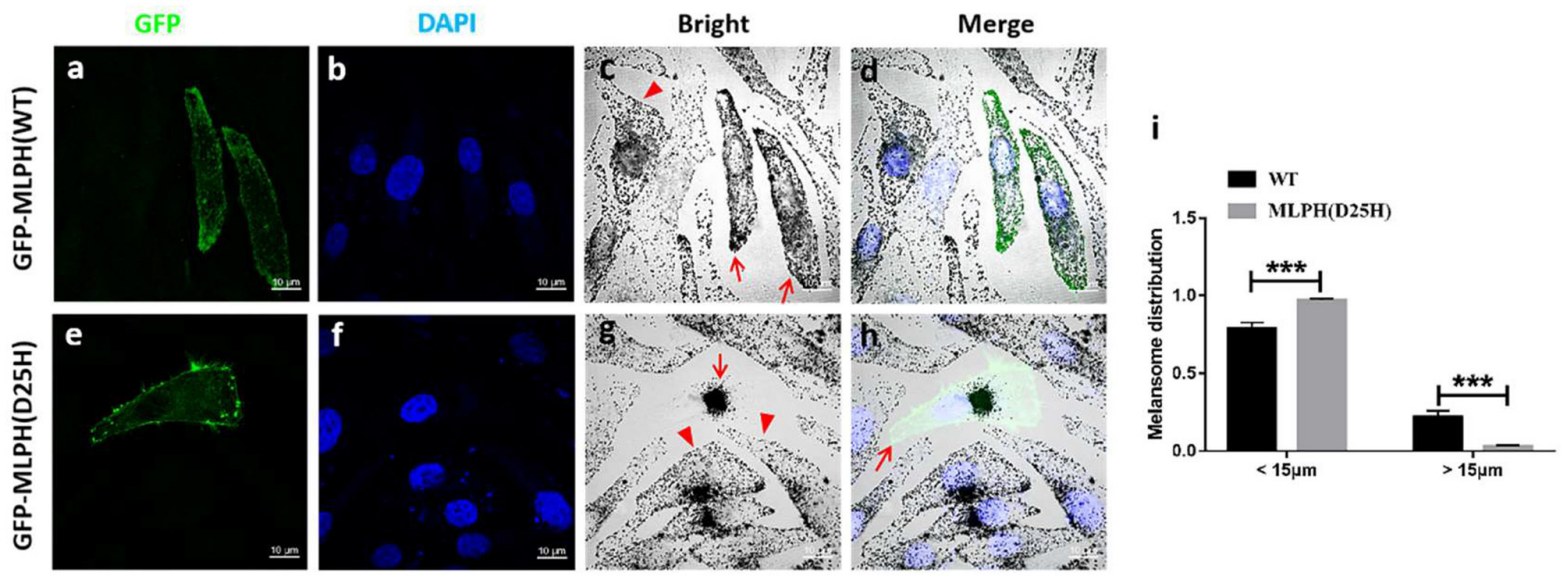
The mutant MLPH (D25H) induces perinuclear melanosome accumulation in MNT1 cell by bright-field microscopy. **(a–h)** Images of transfected cells. Green fluorescence protein (GFP) [**(a,e)**, green] represents the transfected cells. Blue staining represents DAPI-labeled nuclei **(b,f)**. Melanocytes transfected with GFP-MLPH (WT) show the presence of pigmented melanosomes in the cell peripheral [**(c,d)**, red arrows], while melanocytes transfected with GFP-MLPH (D25H) show a perinuclear aggregation of melanosomes [**(g)**, red arrow] and the absence of melanosomes in peripheral region [**(h)**, red arrow]. Cells failed to transfect with GFP-MLPH (WT) [**(c)**, red arrowhead] or GFP-MLPH (D25H) [**(g)**, red arrowheads] show a normal peripheral melanosome distribution. Bars: 10 μm. **(i)** Quantification of melanosome radial profile distribution. Melanosome distribution was quantified using Image J plugin radial profile. The radial melanosome intensity from each cell was quantified by drawing an equally-sized circle around its entire area. Percentage distribution of “perinuclear” melanosome (< 15 μm) and “peripheral” melanosome (> 15 μm) was calculated. In total, 11 cells in each group were analyzed. Quantified data were presented as mean ± standard error of the mean (SEM) using Student's *t*-test. Error bars indicate SEM (***p < 0.001).

**Figure 5:**
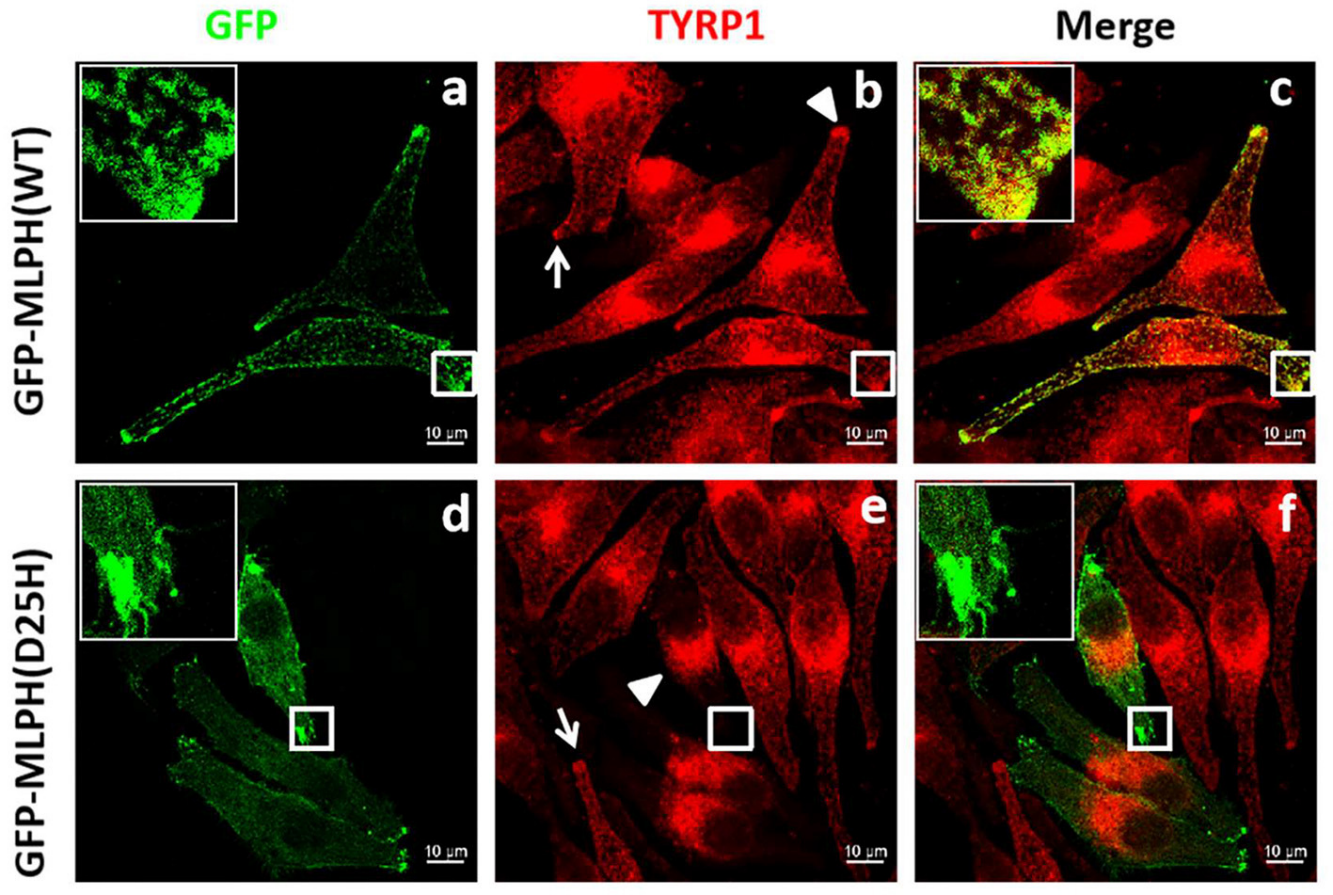
Distribution of TYRP1 in melanocytes transfected with GFP-MLPH (WT) or GFP-MLPH (D25H). **(a–f)** MNT1 cells were transfected with GFP-MLPH (WT) or GFP-MLPH (D25H). Transfection with GFP-MLPH (WT) showed a peripheral distribution of TYRP1 [**(b)**, arrowhead], while transfection with GFP-MLPH (D25H) showed the typical clustering of TYRP1 in the perinuclear region [**(e)**, arrowhead] and lack of the TYRP1 in the cell periphery [**(e)**, rectangle]. GFP-MLPH (WT) colocalized with TYRP1 **(c)** in the cell periphery while GFP-MLPH (D25H) did not **(f)**. Bars: 10 μm.

To evaluate the impact of the D25H mutation on MLPH, the MLPH expression level in the platelets of the patient was determined by Western blotting. HEK293T cells were transfected with Flag-MLPH and our results showed both Flag and MLPH antibodies recognized the same specific band, which confirmed the specificity of the MLPH antibody (Figure 3A). As shown in Figure 3B, the protein levels of MLPH, MYO5A, and RAB27A were not altered in the patients with GS3 compared with an unaffected control. Next, we examined the localization pattern of the MLPH (D25H) protein in MNT-1 cells. We observed that both the WT and mutant MLPH proteins were associated primarily with punctate structures located throughout the cell body and peripheral dendrites (Figures 4a,e, 5a,d), similar to the MLPH (WT) distribution described for melan-a melanocytes (5). Therefore, the D25H mutation did not interfere with the expression and localization of the MLPH protein.

### SHD (D25H)-MLPH Reduced the Interaction With RAB27A

The RAB27A protein binds to the surface of the melanosome and participates in actin-dependent melanosome movement *via* direct interaction with its effector MLPH and indirect interaction with MYO5A (24, 25). To further define whether the D25H substitution compromises the RAB27A-MLPH interaction, an SHD (D25H)-MLPH construct was used to express the D25H mutant. Flag-RAB27A and Myc-tagged SHD (WT), SHD (D25H), or SHD (R35W) were co-transfected into HEK293T cells, respectively. Cell lysates were immunoprecipitated with anti-Flag beads, followed by immunoblotting with anti-Flag and anti-Myc antibodies, respectively (Figure 3C). The results showed that the SHD (D25H)-MLPH variant reduced the binding to RAB27A protein, compared with the control SHD (WT)-MLPH (Figure 3C). As a positive control, the interaction between SHD (R35W)-MLPH and RAB27A was also decreased, as previously reported (8). These data suggest that the conserved D25 residue of MLPH plays role in the interaction with RAB27A.

### MLPH (D25H) Induces Perinuclear Distribution of Melanosomes in Human Melanocytes

It was previously described that mutations in *MLPH* cause the clustered perinuclear distribution observed in leaden mice (26). The pathologic defect in a GS3 patient with the MLPH (R35W) substitution induced aggregation of melanosomes in the perinuclear area of the patient's melanocytes (27). To test whether MLPH (D25H) is disease-causing, light microscopy and immunofluorescence confocal microscopy were used to examine the distribution of melanosomes. Green fluorescence showed the plasmid was successfully transfected into the cells (Figures 4a,e, 5a,d). Both melanocytes transfected with green fluorescence protein (GFP)-MLPH (WT) (Figure 4c, red arrows) and un-transfected cells from the same field of view (Figure 4c, red arrowhead) had a normal distribution of melanosomes at the periphery of the cells. In contrast, melanocytes overexpressed GFP-MLPH (D25H) showed melanosome accumulation in the perinuclei (Figure 4g, red arrow) and a lack of melanosomes in the peripheral dendrites (Figure 4h, red arrow), while those adjacent un-transfected cells exhibited a normal melanosome distribution (Figure 4g, red arrowheads). Quantification of melanosome distribution showed that more melanosomes were clustered in the perinuclear region but less in the periphery (Figure 4i).

Next, we used laser scanning confocal microscopy to study the localization of the melanosome-specific protein TYRP1 (Figures 5b,e), which is involved in the biosynthesis of melanin and the maintenance of melanosome structures (28, 29). In melanocytes transfected with or without GFP-MLPH (WT) (Figure 5b), TYRP1 staining showed a punctate pattern in the cell periphery and dendrites (Figure 5b, white arrowhead and arrow), with a slight aggregation around the nuclei. MLPH colocalized with TYRP1 in the cell's periphery dendritic tip (Figure 5c). By comparison, melanocytes overexpressed GFP-MLPH (D25H) showed a perinuclear accumulation (Figure 5e, white arrowhead) and the dendrites were devoid of TYRP1 staining (Figure 5e, white rectangle). While un-transfected cells displayed a normal localization of TYRP1 (Figure 5e, arrow). However, there was almost no colocalization between the MLPH (D25H) and TYRP1 in the cell periphery (Figure 5f). Taken together, these results demonstrated that the D25H mutation resulted in perinuclear aggregation of melanosomes, suggesting the impeded movement of melanosomes toward the cell periphery, which underlies the pathological effects of the patient with GS3.

## Discussion

All members of the Slp-family proteins share an N-terminal SHD, including two conserved potential a-helical regions (SHD1 and SHD2) often separated by two zinc finger motifs (30). Within this region, SHD1 directly binds to the switch II region of the GTP-bound active form of RAB27A on the melanosome membrane (5, 31). Furthermore, RAB27A-GTP recruits MLPH to the melanosomes through its interaction with both SHD1 and SHD2 (32). Thus, the SHD domain is critical for the formation of the tripartite complex involved in melanosome transport. We here reported a new pathological missense variant c.73G > C (p.D25H) that is located in the SHD domain together with the other four reported variants (Figure 2D). Although the D25H variant did not alter the protein expression level and melanosomal localization, it resulted in the stuck of melanosomes in the perinuclear region of the melanocytes, leading to the clumps of pigment in the patient's hair shafts.

Slp homology domain 1 of melanophilin alone is both necessary and sufficient for high-affinity specific recognition of the GTP-bound form of RAB27A. By contrast, the zinc finger motifs and SHD2 seem to be important for the stabilization of the structure of the SHD or higher affinity RAB27A binding (31). The R35W/F/K mutation in the SHD1 domain prevents it from interacting with RAB27A (8), and R35W introduces melanosome aggregation in cultured melanocytes (27). Similarly, the D25H mutation is located in the SHD1 domain, leading to melanosome aggregation in transfected melanocytes. Furthermore, we confirmed the decreased interaction between SHD (D25H)-MLPH and RAB27A, similarly to SHD (R35W)-MLPH and RAB27A, suggesting that both D25H and R35W may have a similar mechanism in disrupting the MLPH-RAB27A interaction that blocks the melanosome transport to the cell periphery where the melanosome releases its melanin content. Our findings not only expand the mutational spectrum of MLPH but also emphasize the importance of the SHD1 domain in mediating melanosome transport.

## Data Availability Statement

The datasets presented in this article are not readily available because of ethical/privacy restrictions. Requests to access the datasets should be directed to the corresponding authors.

## Ethics Statement

This study was approved by the Ethics Committees of Beijing Tongren Hospital and Beijing Children's Hospital, Capital Medical University. Written informed consent was obtained from the participant for the publication of any potentially identifiable data or images.

## Author Contributions

AW and WL designed the study and finalized the manuscript. QH and YY performed the experiments, analyzed the data, and wrote the manuscript. JG, TZ, ZQ, and XY provided technical support. All authors approved the submitted version of the manuscript.

## Funding

This work was partially supported by grants from the Ministry of Science and Technology of China [2019YFA0802104 (WL)] and from the National Natural Science Foundation of China [(82173447 (AW), 31830054 (WL), and 31900496 (YY)].

## Conflict of Interest

The authors declare that the research was conducted in the absence of any commercial or financial relationships that could be construed as a potential conflict of interest.

## Publisher's Note

All claims expressed in this article are solely those of the authors and do not necessarily represent those of their affiliated organizations, or those of the publisher, the editors and the reviewers. Any product that may be evaluated in this article, or claim that may be made by its manufacturer, is not guaranteed or endorsed by the publisher.
