# Supplementary figures and images for: Identification of a Novel MLPH Missense Mutation in a Chinese Griscelli Syndrome 3 Patient
Source: Front Med (Lausanne). 2022 May 6;9:896943. doi: 10.3389/fmed.2022.896943 (PMC9120966; doi:10.3389/fmed.2022.896943)

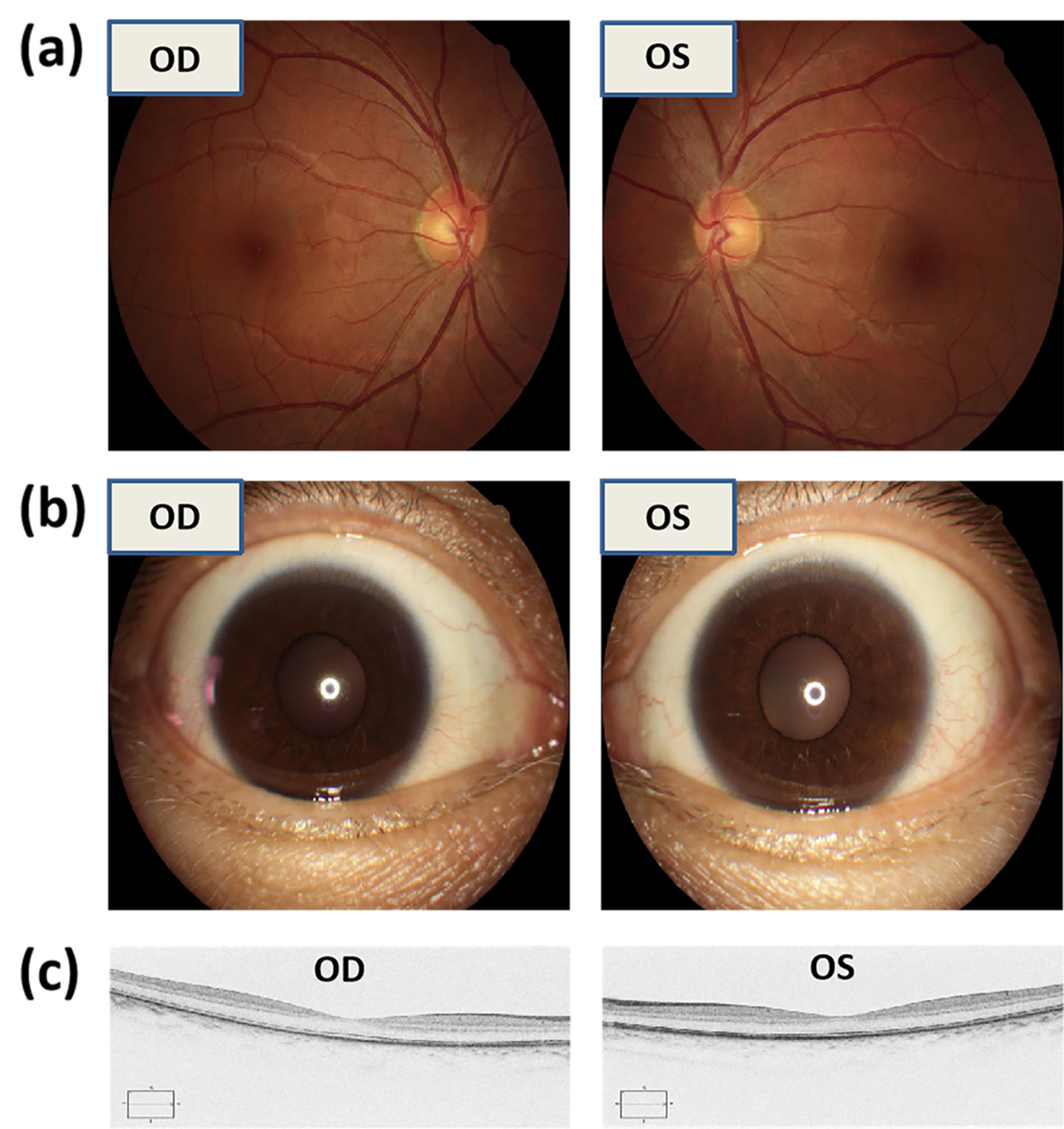

Supplement: Supplementary file 1 [file Image_1.tif]
